# Supplementary material for: Gut microbiota impact on the peripheral immune response in non-alcoholic fatty liver disease related hepatocellular carcinoma
Source: Nat Commun. 2021 Jan 8;12:187. doi: 10.1038/s41467-020-20422-7 (PMC7794332; doi:10.1038/s41467-020-20422-7)
Supplement: Supplementary file 1 — Supplementary information [file 41467_2020_20422_MOESM1_ESM.pdf]

SUPPLEMENTARY FIGURES

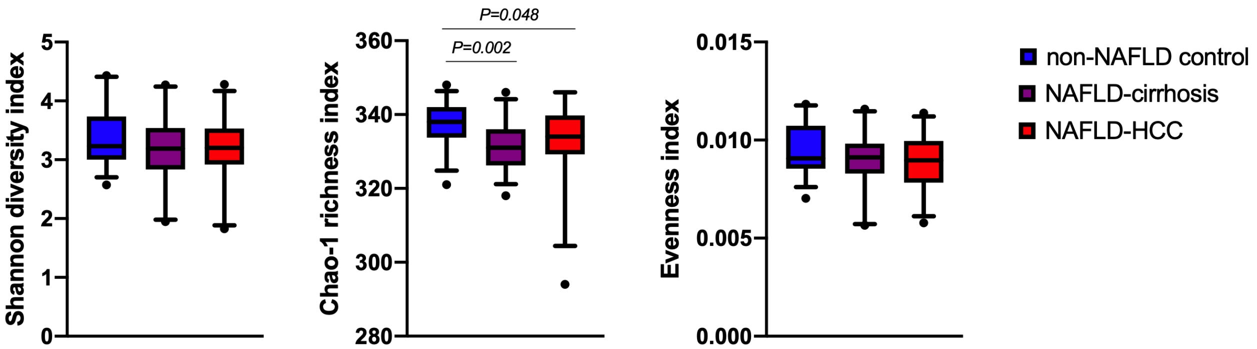

Supplementary Figure 1. Alpha diversity indices of non-NAFLD control, NAFLD-cirrhosis and NAFLD-HCC faecal samples

Alpha diversity based on Shannon diversity, Chao-1 richness and Evenness indices of non-NAFLD control, NAFLD-cirrhosis and NAFLD-HCC faecal samples. Sample size is n=30 non-NAFLD control, n=28 NAFLD-cirrhosis, n=32 NAFLD-HCC as biologically independent samples. Box plots indicate median (middle line), 25th, 75th percentile (box) and 5th and 95th percentile (whiskers) as well as outliers (single points). *P* calculated are calculated by Kruskal-Wallis for 3 group comparison and Dunn's test for 2 group comparison. Source data are provided as a Source Data file.

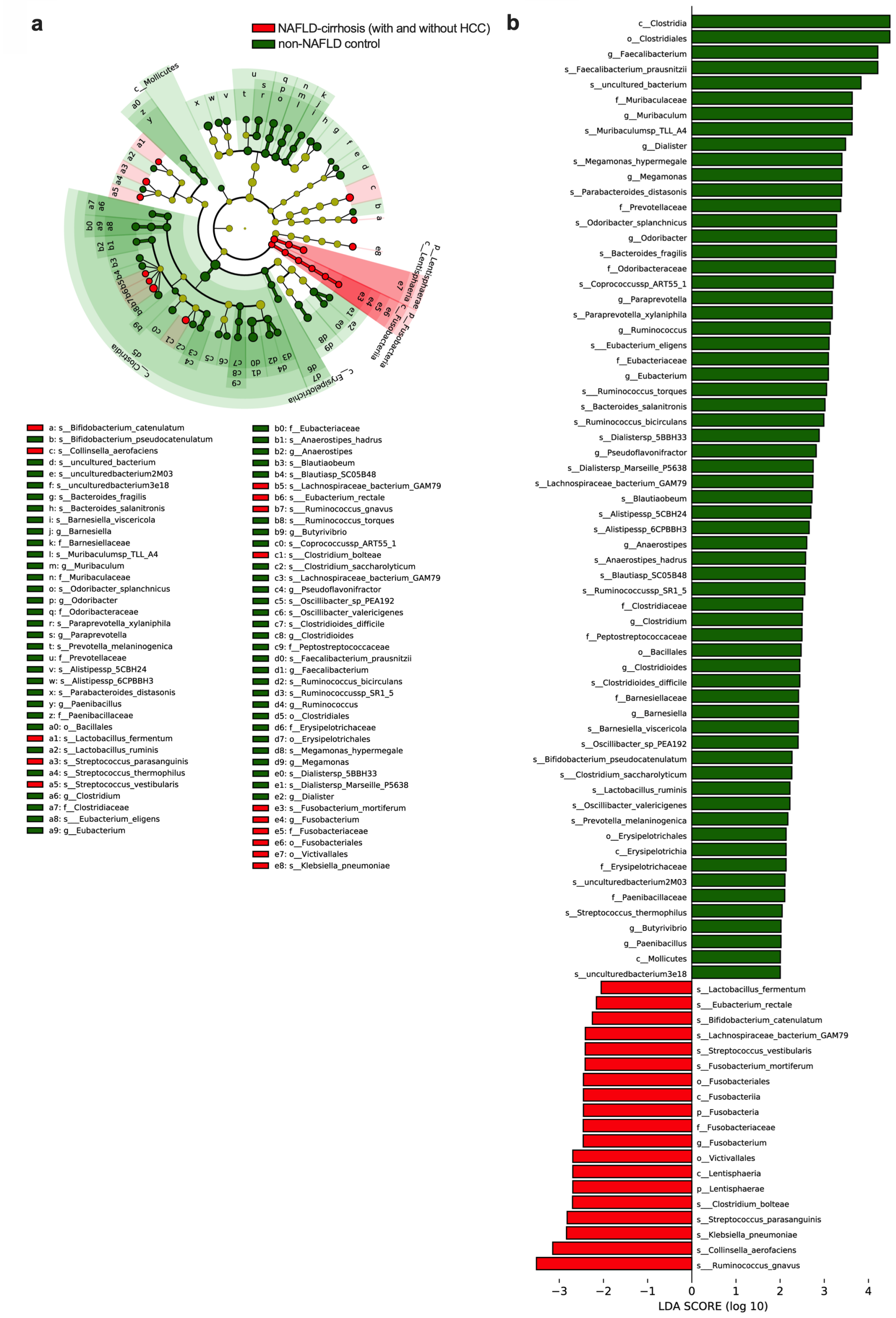

Supplementary Figure 2. Microbiome composition of all patients with NAFLD-cirrhosis (with and without HCC) and non-NAFLD controls.

a) Cladogram displaying taxonomic tree of differentially abundant taxa in whole population of NAFLD-cirrhosis (with and without HCC) compared to non-NAFLD controls. **b)** Histogram displaying linear discriminant analysis (LDA) scores for NAFLD-cirrhosis (with and without HCC) compared to non-NAFLD control calculated by LEfSe analysis. Only taxa with LDA > 2 or LDA < -2 are shown. For a-b) sample size is n=30 non-NAFLD control, n=60 for NAFLD-cirrhosis (with and without HCC) as biologically independent samples.

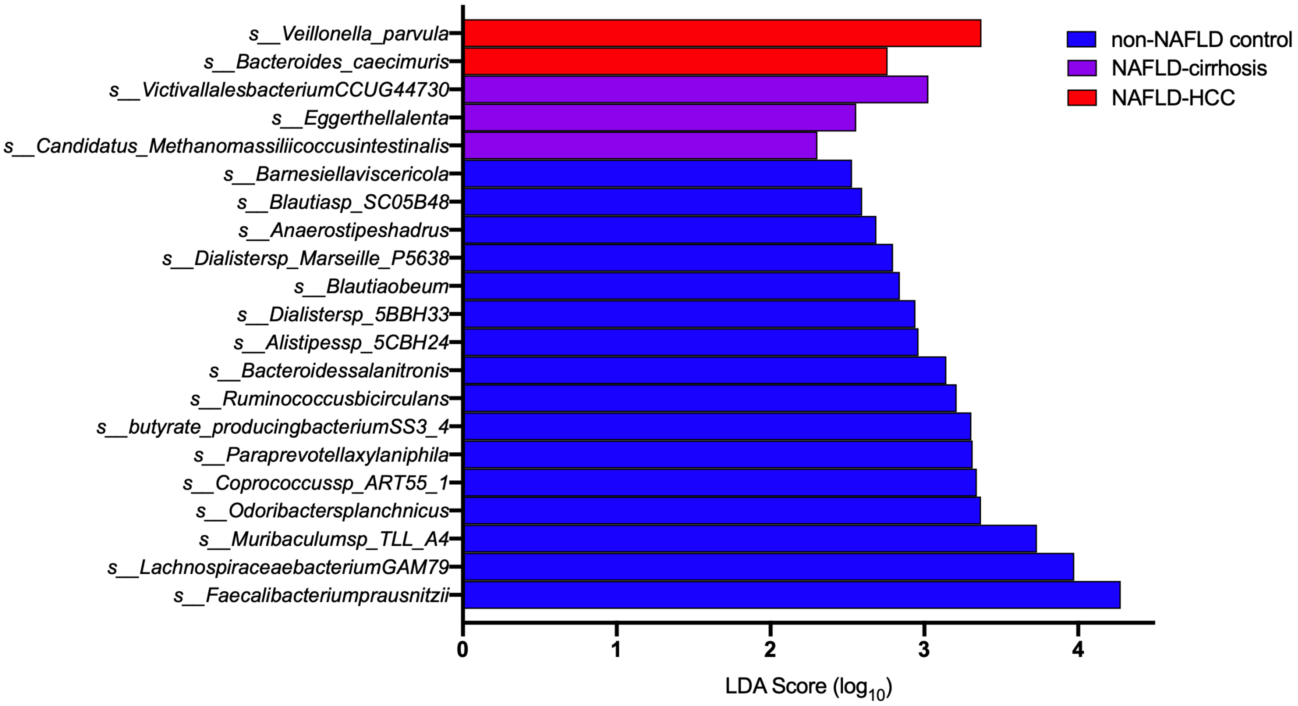

Supplementary Figure 3. Microbiome composition at species level in faecal samples from non-NAFLD controls, NAFLD-cirrhosis and NAFLD-HCC subjects

Histogram displaying linear discriminant analysis (LDA) scores for non-NAFLD control, NAFLD-cirrhosis and NAFLD-HCC faecal samples calculated by LEfSe analysis. Sample size is n=30 non-NAFLD control, n=28 NAFLD-cirrhosis, n=32 NAFLD-HCC as biologically independent samples Only species with LDA log10 > 2.5 are shown. Source data are provided as a Source Data file.

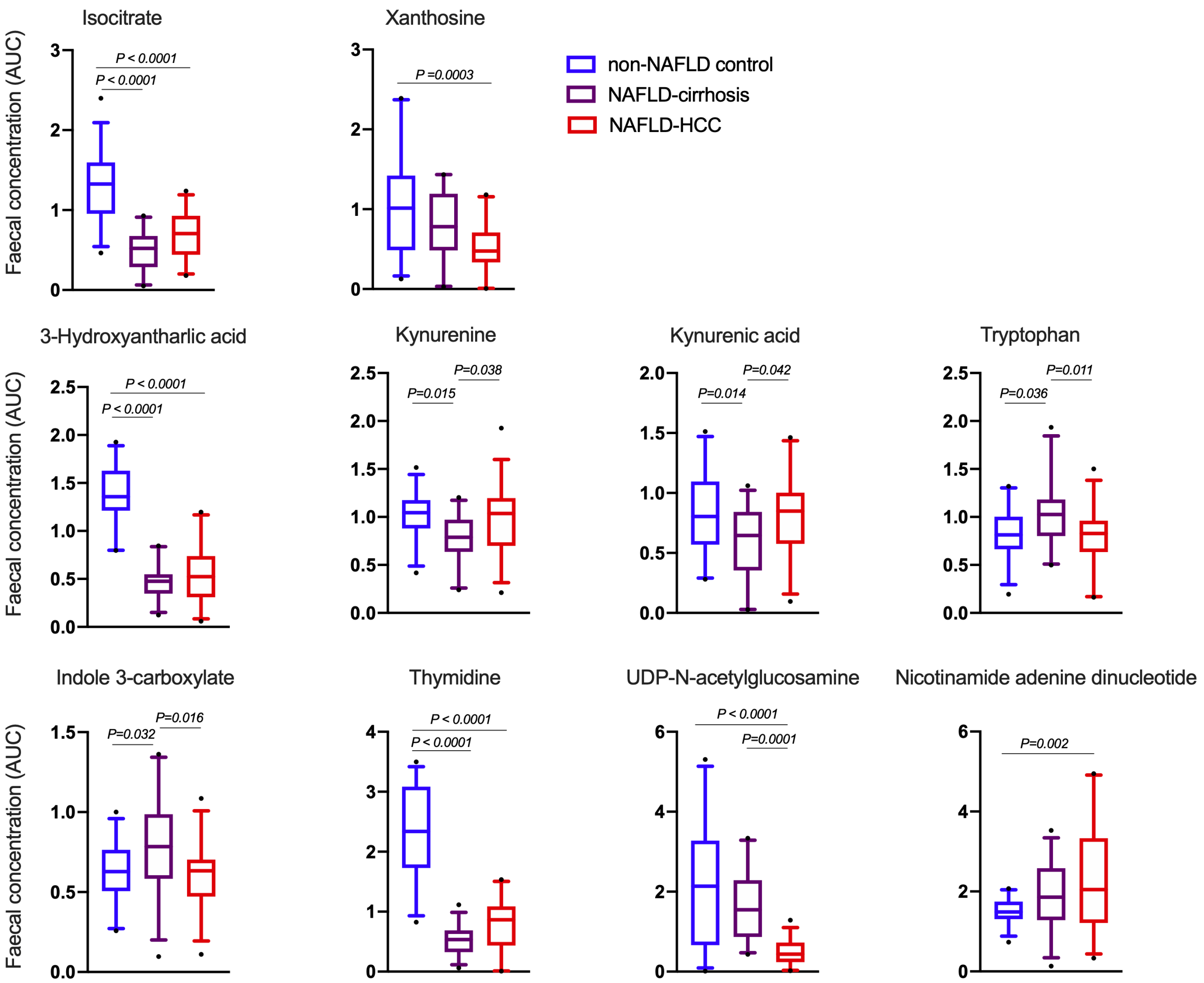

Supplementary Figure 4. Faecal metabolites where differences in concentration were seen in NAFLD-HCC, NAFLD-cirrhosis and non-NAFLD control groups

Faecal metabolites were differences in concentration were seen across groups. Data represented as area under the curve (AUC) relative to pooled sample. Sample size is n=30 non-NAFLD control, n=28 NAFLD-cirrhosis, n=32 NAFLD-HCC as biologically independent samples. Box plots indicate median (middle line), 25th, 75th percentile (box) and 5th and 95th percentile (whiskers) as well as outliers (single points). *P* values are calculated by one-way ANOVA for 3 group comparison and Tukey's test for 2 group comparison. All data is shown in Supplementary Table 4. Source data are provided as a Source Data file.

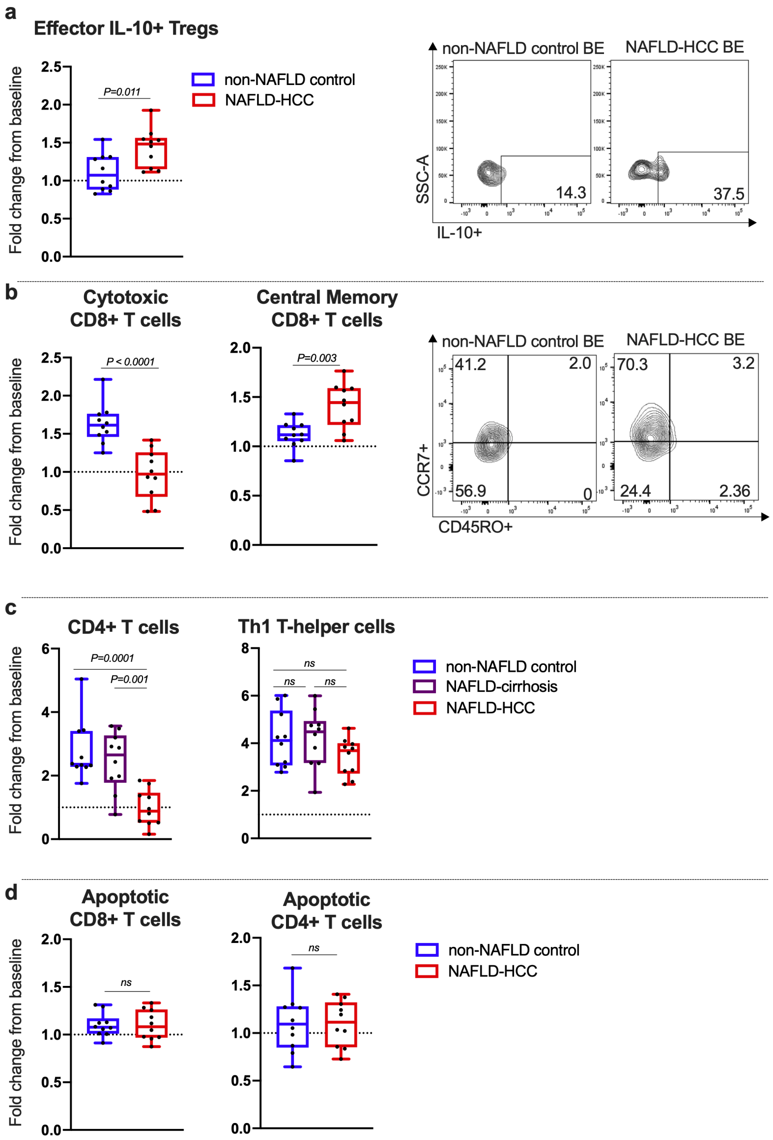

Supplementary Figure 5. NAFLD-HCC bacterial extract (BE) induces effector IL-10+ Tregs, attenuates the expansion of cytotoxic CD8+ cells in addition to mediating a phenotype switch to central memory CD8+ T cells. Additionally, NAFLD-HCC BE attenuates the expansion of total CD4+ T cell populations, but not T-helper populations in non-NAFLD control PBMCs. Attenuated expansion of CD8+ and CD4+ T cell populations induced by NAFLD-HCC bacterial extract (BE) is not a result of apoptosis.

Fold change and representative flow cytometry plots of **a)** effector IL-10+ Tregs (CD3+CD4+CD25+Foxp3+CD45RO+IL-10+) in response to non-NAFLD control and NAFLD-HCC BE **b)** cytotoxic CD8+ T cells (CD3+CD8+CCR7-CD45RO-) and central memory CD8+ T cells (CD3+CD8+CCR7+CD45RO+) in response to non-NAFLD control and NAFLD-HCC BE and **c)** total CD4+ lymphocytes (CD3+CD4+) and Th1 T-helper cells (CD3+CD4+Tbet+) in response to non-NAFLD control, NAFLD-cirrhosis and NAFLD-HCC BE. Fold change of **d)** total CD8+Annexin+ lymphocytes and total CD4+Annexin+ lymphocytes in response to non-NAFLD control and NAFLD-HCC BE. All data is represented as % of viable CD3+ lymphocytes normalised to baseline measurements. Baseline immune response is represented as dashed line. For a, b and d) sample size is n=10 non-NAFLD control and n=10 NAFLD-HCC as biologically independent samples; for c) sample size is n=10 non-NAFLD control, n=10 NAFLD-cirrhosis, n=10 NAFLD-HCC as biologically independent samples. Box plots indicate median (middle line), 25th, 75th percentile (box) and 5th and 95th percentile (whiskers) as well as outliers (single points). For a, b and d) *P* values are calculated with two-tailed t-test; for c) *P* values are calculated by one-way ANOVA for 3 group comparison and Tukey's test for 2 group comparison. All data is shown in Supplementary Table 1. Source data are provided as a Source Data file.

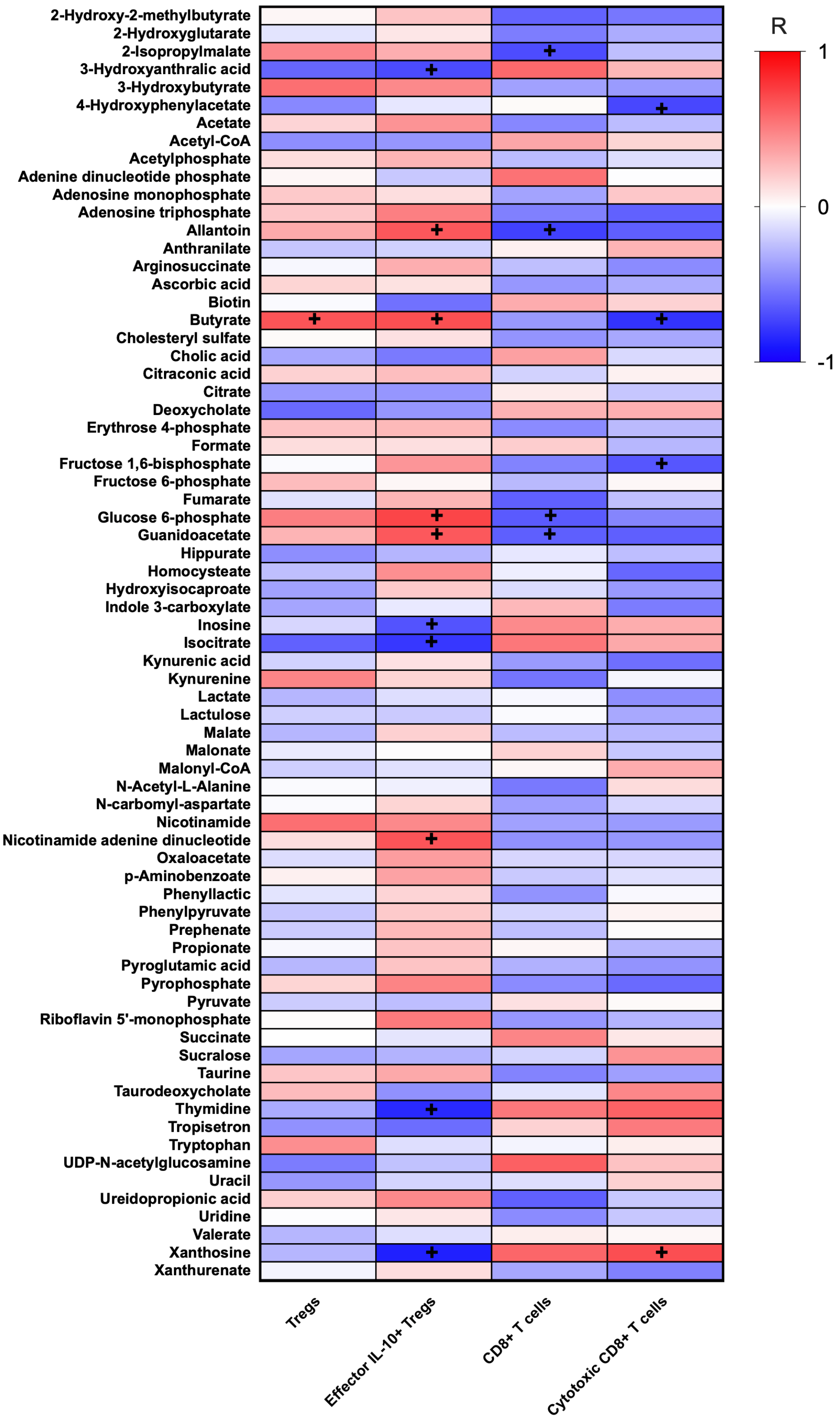

Supplementary Figure 6. Correlations between faecal metabolites and T cell immune responses measured *ex vivo*.

Heatmap of Spearman's rank correlation of all metabolites measured in faeces and T cell immune responses measured *ex vivo*. Colour legend represents correlation coefficient (R). "+" denotes significant correlation ($P < 0.05$) are shown after Benjamini-Hochberg correction. Source data are provided as a Source Data file.

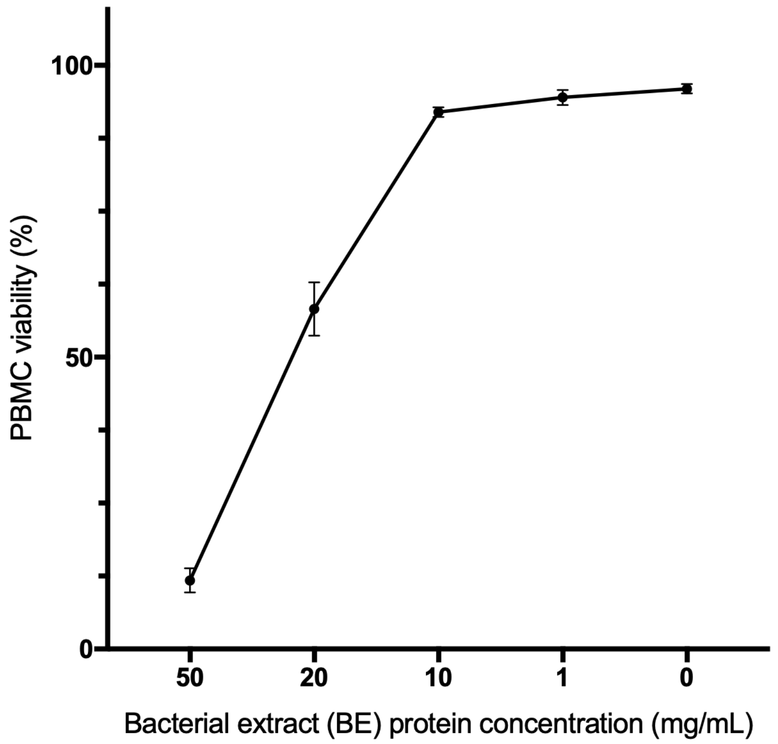

Supplementary Figure 7. Increase in bacterial extract (BE) dose impacts on PBMC viability in *ex vivo* model

Percentage of viable PBMCs, following incubation with bacterial extract (BE) (n=4) demonstrating significant reduction in PBMC viability (< 90%) at doses exceeding 10mg/mL. Data shown as mean (±SD). PBMC viability was assessed with light microscopy using 20 μL of PBMC sample mixed with 20 μL of 0.4% trypan blue solution. Source data are provided as a Source Data file.

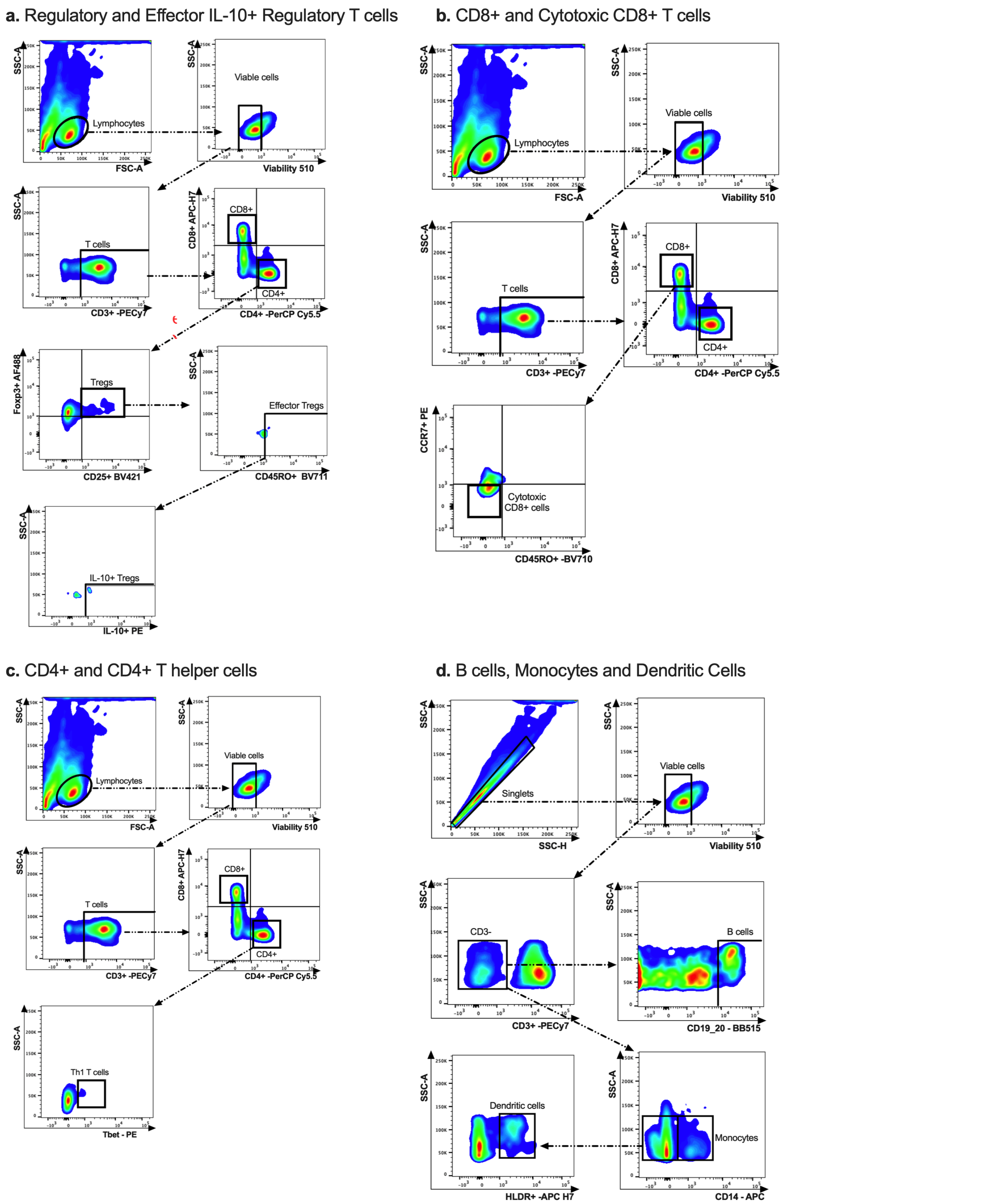

Supplementary Figure 8. Gating strategy for flow cytometry analysis of target cell populations in peripheral blood mononuclear cells

Gating strategy for **a)** CD4+ T cells (CD3+CD4+), Tregs (CD3+CD4+CD25+Foxp3+) and Effector Tregs (CD3+CD4+CD25+Foxp3+CD45RO+IL-10+) from viable lymphocyte populations **b)** CD8+ T cells (CD3+CD8+), cytotoxic CD8+ T cells (CD3+CD8+CCR7-CD45RO-) and central memory CD8+ T cells (CD3+CD8+CCR7+CD45RO+) from viable lymphocyte populations **c)** CD4 Th1 cells (CD3+CD4+Tbet+) from viable lymphocyte populations and **d)** B cells (CD3-CD19+CD20+), Monocytes (CD3-CD14+) and Dendritic cells (CD3-CD14-HLADR+) from viable singlets.

SUPPLEMENTARY TABLES

Supplementary Table 1. Comparison of immune cell populations in NAFLD-HCC, NAFLD-cirrhosis and non-NAFLD control cohorts.
Sample size for PBMC (baseline) data: n=30 non-NAFLD control, n=28 NAFLD-cirrhosis, n=32 NAFLD-HCC as biologically independent samples. Sample size for PBMC (fold change from baseline) data: n=10 non-NAFLD control, n=10 NAFLD-cirrhosis, n=10 NAFLD-HCC as biologically independent samples. Data is shown as mean ± standard error of mean (SEM). *P* values calculated by two tailed t-test for two group comparison or *P* values calculated by one-way ANOVA for 3 group comparison with Tukey's post hoc test for 2 group comparisons. $P < 0.05$ considered statistically significant.

Variable	non-NAFLD control Mean (±SEM)	NAFLD-cirrhosis Mean (±SEM)	NAFLD-HCC Mean (±SEM)	*P*-value (two tailed t-test or one-way ANOVA)	*P*-value non-NAFLD control vs NAFLD-cirrhosis	*P*-value non-NAFLD control vs NAFLD-HCC	*P*-value NAFLD-cirrhosis vs NAFLD-HCC
PBMCs (baseline)							
Baseline Tregs (%)	0.620(±0.041)	0.480(±0.044)	1.380(±0.068)	<0.0001	0.1727	<0.0001	<0.0001
Baseline CD8+ T cells (%)	19.500(±0.539)	19.750(±0.670)	16.760(±1.078)	0.0174	0.9754	0.0472	0.0307
PBMCs (fold change from baseline)							
Tregs	1.000(±0.187)	1.120(±0.191)	2.140(±0.283)	0.0021	0.9244	0.0036	0.0092
Effector IL-10+ Tregs	1.110(±0.078)	_	1.430(±0.081)	0.0105	_	_	_
CD8+ T cells	0.987(±0.046)	1.017(±0.043)	0.570(±0.129)	0.0010	0.9651	0.0039	0.0020
Cytotoxic CD8+ T cells	1.630(±0.083)	_	0.970(±0.103)	<0.0001	_	_	_
Central memory CD8+ T cells	1.120(±0.041)	_	1.410(±0.073)	0.0027	_	_	_
CD4+ T cells	2.780(±0.300)	2.450(±0.293)	0.980(±0.180)	<0.0001	0.6545	0.0001	0.0014
Th1 T-helper cells	4.170(±0.377)	4.200(±0.380)	3.430(±0.249)	0.2130	0.9979	0.2907	0.2638
Monocytes	2.170(±0.105)	2.220(±0.054)	1.750(±0.108)	0.0022	0.9231	0.0093	0.0036
B cells	1.330(±0.060)	1.340(±0.114)	0.970(±0.066)	0.0055	0.9962	0.0140	0.0114

Supplementary Table 2. Comparison of relative taxonomic abundance at phylum, family and species level in NAFLD-HCC, NAFLD-cirrhosis and non-NAFLD control cohorts.

Sample size is n=30 non-NAFLD control, n=28 NAFLD-cirrhosis, n=32 NAFLD-HCC as biologically independent samples. Data is shown as mean % ± standard error of mean (SEM). P values calculated by Kruskal-Wallis for 3 group comparison and Dunn's post hoc test for 2 group comparisons. $P < 0.05$ considered statistically significant.

Taxonomy (relative abundance, %)	non-NAFLD control Mean (±SEM)	NAFLD-cirrhosis Mean (±SEM)	NAFLD-HCC Mean (±SEM)	*P*-value (Kruskal-Wallis test)	*P*-value non-NAFLD control vs NAFLD-cirrhosis	*P*-value non-NAFLD control vs NAFLD-HCC	*P*-value NAFLD-cirrhosis vs NAFLD-HCC
Phylum level							
p_Actinobacteria	2.170E+00(±5.669E-01)	3.431E+00(±9.220E-01)	4.911E+00(±1.554E+00)	0.2110	0.5618	0.2827	>0.9999
p_Verrucomicrobia	3.949E+00(±2.008E+00)	9.028E+00(±3.043E+00)	3.405E+00(±1.439E+00)	0.1502	0.7706	>0.9999	0.2124
p_Proteobacteria	3.379E+00(±4.225E-01)	6.693E+00(±1.410E+00)	8.678E+00(±2.042E+00)	0.0412	0.2753	0.0329	0.6168
p_Firmicutes	3.636E+01(±2.478E+00)	3.200E+01(±2.432E+00)	3.303E+01(±2.717E+00)	0.7787	0.4661	0.6206	0.9563
p_Bacteroidetes	5.322E+01(±2.742E+00)	5.125E+01(±3.171E+00)	4.751E+01(±2.924E+00)	0.3698	0.8879	0.3488	0.6440
Family level							
f__Erysipelotrichaceae	1.820E-03(±1.729E-04)	1.630E-03(±1.672E-04)	1.180E-03(±1.768E-04)	0.0276	0.7267	0.0252	0.1644
f__Lactobacillaceae	2.661E-03(±1.829E-03)	1.144E-03(±4.594E-04)	1.315E-03(±9.272E-04)	0.6352	0.6653	0.7125	0.9941
f__Eggerthellaceae	1.769E-03(±3.449E-04)	2.124E-03(±4.218E-04)	1.369E-03(±1.770E-04)	0.2602	0.7298	0.6447	0.2310
f__Peptostreptococcaceae	2.240E-03(±2.081E-04)	2.550E-03(±1.746E-04)	2.480E-03(±1.681E-04)	0.4687	0.4755	0.6198	0.9613
f__Clostridiaceae	2.367E-03(±2.165E-04)	1.858E-03(±2.508E-04)	2.276E-03(±4.082E-04)	0.4846	0.4951	0.9766	0.6107
f__Desulfovibrionaceae	4.128E-03(±1.361E-03)	2.905E-03(±8.395E-04)	2.912E-03(±1.156E-03)	0.6898	0.7435	0.7285	>0.9999
f__Selenomonadaceae	7.230E-03(±3.341E-03)	1.809E-03(±9.264E-04)	1.001E-03(±6.314E-04)	0.0657	0.1581	0.0760	0.9573
f__Muribaculaceae	1.095E-02(±9.494E-04)	6.460E-03(±3.969E-04)	8.510E-03(±7.955E-04)	0.0005	0.0003	0.0627	0.1481
f__Coriobacteriaceae	3.570E-03(±2.008E-05)	4.676E-03(±4.725E-04)	4.119E-03(±2.652E-04)	0.0445	0.0340	0.3969	0.3996
f__Acidaminococcaceae	3.599E-03(±1.203E-03)	7.862E-03(±2.153E-03)	6.011E-03(±1.821E-03)	0.2434	0.2161	0.5872	0.7383
f__Rikenellaceae	6.407E-02(±1.028E-02)	9.350E-02(±2.525E-02)	5.572E-02(±9.389E-03)	0.2255	0.4077	0.9248	0.2208
f__Streptococcaceae	1.975E-03(±3.936E-04)	1.519E-02(±1.347E-02)	5.005E-03(±1.639E-03)	0.4278	0.4253	0.9525	0.5908
f__Veillonellaceae	9.673E-03(±3.230E-03)	6.117E-03(±2.561E-03)	9.206E-03(±3.155E-03)	0.6770	0.6940	0.9932	0.7523
f__Odoribacteraceae	1.315E-02(±1.315E-03)	8.979E-03(±7.181E-04)	1.068E-02(±9.192E-04)	0.0202	0.0177	0.1982	0.4723
f__Acidobacteriaceae	7.312E-05(±1.089E-05)	6.445E-05(±1.272E-05)	6.172E-05(±1.275E-05)	0.7845	0.8739	0.7797	0.9863
f__Prevotellaceae	1.435E-02(±1.315E-03)	9.850E-03(±7.181E-04)	1.143E-02(±9.192E-04)	0.0100	0.0086	0.1064	0.5230
f__Bifidobacteriaceae	8.686E-03(±2.209E-03)	1.440E-02(±4.760E-03)	1.728E-02(±5.667E-03)	0.3869	0.6549	0.3626	0.8946
f__Oscillospiraceae	1.600E-01(±1.187E-02)	1.520E-01(±1.512E-02)	1.182E-01(±9.192E-03)	0.0341	0.8895	0.0384	0.1236
f__Tannerellaceae	3.384E-02(±4.636E-03)	2.931E-02(±6.225E-03)	2.991E-02(±5.227E-03)	0.8122	0.8276	0.8587	0.9966
f__Enterobacteriaceae	2.992E-02(±2.739E-03)	3.022E-02(±2.835E-03)	4.290E-02(±4.419E-03)	0.0124	0.9980	0.0247	0.0327
f__Akkermansiaceae	2.900E-02(±1.585E-02)	6.558E-02(±2.260E-02)	2.516E-02(±9.698E-03)	0.1711	0.2710	0.9844	0.1949
f__Eubacteriaceae	5.472E-02(±5.477E-03)	9.500E-02(±9.449E-03)	6.772E-02(±4.596E-03)	0.0002	0.0002	0.3404	0.0128
f__Lachnospiraceae	9.699E-02(±1.200E-02)	6.798E-02(±7.877E-03)	8.325E-02(±1.047E-02)	0.1576	0.1329	0.6094	0.5548
f__Ruminococcaceae	1.225E-01(±1.469E-02)	9.329E-02(±1.401E-02)	1.014E-01(±1.438E-02)	0.3460	0.3406	0.5459	0.9167
f__Bacteroidaceae	4.903E-01(±3.524E-02)	4.545E-01(±3.916E-02)	5.182E-01(±3.559E-02)	0.4731	0.7759	0.8479	0.4400
Species level							
s_Bacteroides caecimuris	1.238E-01(±2.686E-02)	2.210E-01(±4.554E-02)	3.531E-01(±4.918E-02)	<0.0001	0.0648	<0.0001	0.0162
s_Bacteroides xylanisolvens	7.421E-01(±1.026E-01)	2.028E+00(±4.721E-01)	1.702E+00(±2.710E-01)	0.0075	0.0146	0.0223	>0.9999
s_Ruminococcus gnavus	8.315E-02(±1.602E-02)	3.486E-01(±8.011E-02)	1.059E+00(±2.606E-01)	<0.0001	0.0474	<0.0001	0.0382
s_Veillonella parvula	9.585E-03(±2.762E-03)	3.081E-02(±2.069E-02)	3.960E-01(±1.208E-01)	0.0019	>0.9999	0.0181	0.0032
s_Clostridium bolteae	8.387E-02(±1.569E-02)	1.657E-01(±2.597E-02)	3.381E-01(±5.137E-02)	<0.0001	0.0153	<0.0001	0.0096

Supplementary Table 3. Comparison of relative abundance of SCFA genes in NAFLD-HCC, NAFLD-cirrhosis and non-NAFLD control cohorts.
Sample size is n=30 non-NAFLD control, n=28 NAFLD-cirrhosis, n=32 NAFLD-HCC as biologically independent samples. Data is shown as mean RPKM ± standard error of mean (SEM). *P* values calculated by Kruskal-Wallis for 3 group comparison and Dunn's post hoc test for 2 group comparisons. $P < 0.05$ considered statistically significant. RPKM; Reads per kilobase of transcript, per million mapped reads.

SCFA synthesis gene (relative abundance, RPKM)	non-NAFLD control Mean (±SEM)	NAFLD-cirrhosis Mean (±SEM)	NAFLD-HCC Mean (±SEM)	*P*-value (Kruskal-Wallis test)	*P*-value non-NAFLD control vs NAFLD-cirrhosis	*P*-value non-NAFLD control vs NAFLD-HCC	*P*-value NAFLD-cirrhosis vs NAFLD-HCC
Acetate synthesis pathway							
pta (K00625)	74.180(±5.914)	79.910(±8.895)	97.250(±5.008)	0.0195	>0.9999	0.0464	0.0491
ackA (K00925)	79.380(±6.748)	87.470(±9.740)	88.160(±6.323)	0.6622	0.7410	0.6859	0.9977
Butyrate synthesis							
ptb (K00634)	37.110(±1.387)	38.170(±3.997)	54.890(±4.503)	0.0054	>0.9999	0.0322	0.0092
butK (K00929)	43.030(±4.827)	49.610(±6.064)	47.880(±6.267)	0.7111	0.7091	0.8186	0.9757
bcd (K00248)	4.908(±0.429)	6.097(±1.120)	4.717(±0.936)	0.7158	0.6109	0.9864	0.5053
Acetyl-CoA synthesis from formate							
fdh (K00125)	0.474(±0.241)	0.223(±0.096)	0.222(±0.156)	0.5129	0.5888	0.5642	>0.9999
fhs (K01938)	66.670(±4.802)	74.240(±9.538)	73.640(±5.795)	0.6875	0.7230	0.7452	0.9979
metF (K00297)	79.040(±6.837)	98.460(±7.977)	99.000(±8.238)	0.1208	0.1946	0.1583	0.9987
Acetate and butyrate synthesis							
pdhb (K00162)	6.095(±3.370)	18.540(±6.554)	7.000(±2.210)	0.0829	0.1103	0.9872	0.1406
tesA (K10804)	2.623(±0.240)	2.509(±0.925)	7.745(±3.295)	0.1290	0.9992	0.1908	0.1884
tesB (K10805)	2.623(±0.240)	2.509(±0.302)	2.645(±0.221)	0.9224	0.9475	0.9978	0.9239
ybgC (K07107)	34.290(±4.656)	45.100(±4.709)	44.700(±3.829)	0.1485	0.2061	0.2088	0.9977
yciA (K10806)	35.586(±3.365)	32.550(±1.146)	40.700(±3.829)	0.1784	0.7778	0.4694	0.1616
Priopionate synthesis							
frd (K00244)	6.778(±1.003)	9.832(±2.561)	15.040(±2.657)	0.0209	>0.9999	0.0489	0.0525
sdh (K00239)	23.940(±3.120)	38.300(±6.299)	29.190(±3.074)	0.0683	0.0569	0.6537	0.2953
sucC (K01903)	10.650(±1.325)	17.400(±2.372)	22.930(±2.505)	0.0006	0.0793	0.0003	0.1694
fum (K01677)	31.480(±3.796)	21.370(±3.821)	27.550(±4.322)	0.2183	0.1931	0.7624	0.5255
mdh (K00024)	121.600(±9.906)	144.900(±9.700)	136.000(±8.961)	0.2320	0.2103	0.5243	0.7871
pycA (K01959)	71.030(±6.666)	74.750(±7.618)	113.100(±8.961)	0.0035	>0.9999	0.0096	0.0134
pcc (K01966)	19.690(±3.945)	28.920(±6.374)	21.940(±2.240)	0.3125	0.3071	0.9266	0.4960
mut (K01847)	17.500(±5.848)	37.590(±10.112)	17.320(±4.218)	0.0701	0.1136	0.9998	0.1023

Supplementary Table 4. Comparison of metabolite levels faeces and serum of NAFLD-HCC, NAFLD-cirrhosis and non-NAFLD control cohorts.
Sample size is n=30 non-NAFLD control, n=28 NAFLD-cirrhosis, n=32 NAFLD-HCC as biologically independent samples. Data is shown as mean ± standard error of mean (SEM). *P* values calculated by one-way ANOVA for 3 group comparison and Tukey's post hoc test for 2 group comparisons. $P < 0.05$ considered statistically significant.

Metabolite concentration	non-NAFLD control Mean (±SEM)	NAFLD-cirrhosis Mean (±SEM)	NAFLD-HCC Mean (±SEM)	*P*-value (one-way ANOVA)	*P*-value non-NAFLD control vs NAFLD-cirrhosis	*P*-value non-NAFLD control vs NAFLD-HCC	*P*-value NAFLD-cirrhosis vs NAFLD-HCC
Serum Short Chain Fatty Acids (μM)							
Butyrate	2.750(±0.236)	2.962(±0.254)	3.819(±0.237)	0.0049	0.8157	0.0058	0.0378
Formate	9.530(±0.307)	9.872(±0.327)	10.640(±0.554)	0.1597	0.8406	0.1489	0.4095
Malonate	3.233(±0.219)	4.559(±0.373)	5.397(±0.366)	<0.0001	0.0164	<0.0001	0.1733
Valerate	2.460(±0.164)	2.860(±0.198)	2.980(±0.182)	0.1081	0.2821	0.1051	0.8874
Propionate	0.520(±0.050)	0.424(±0.051)	1.010(±0.154)	0.0002	0.7913	0.0027	0.0003
Acetate	16.980(±1.627)	15.790(±1.889)	22.000(±2.121)	0.0519	0.9014	0.1465	0.0610
Faecal metabolites (AUC)							
Oxaloacetate	0.921(±0.096)	0.510(±0.067)	1.943(±0.202)	<0.0001	0.0511	0.0025	<0.0001
Acetylphosphate	0.860(±0.042)	0.960(±0.055)	1.170(±0.055)	0.0001	0.3717	0.0001	0.0139
Isocitrate	1.305(±0.077)	0.489(±0.047)	0.683(±0.054)	<0.0001	<0.0001	<0.0001	0.0683
Xanthosine	1.040(±0.121)	0.802(±0.084)	0.521(±0.058)	0.0004	0.1682	0.0003	0.0786
Thymidine	2.351(±0.137)	0.527(±0.046)	0.780(±0.073)	<0.0001	<0.0001	<0.0001	0.1453
UDP-N-acetylglucosamine	2.278(±0.287)	1.667(±0.169)	0.484(±0.053)	<0.0001	0.0744	<0.0001	0.0001
Nicotinamide adenine dinucleotide	1.497(±0.056)	1.974(±0.157)	2.331(±0.236)	0.0031	0.1343	0.0021	0.3081
3-Hydroxyanthralic acid	1.378(±0.054)	0.467(±0.035)	0.548(±0.056)	<0.0001	<0.0001	<0.0001	0.4995
2-Hydroxy-2-methylbutyrate	0.881(±0.042)	0.932(±0.061)	0.814(±0.073)	0.3940	0.8311	0.7114	0.3648
2-Hydroxyglutarate	0.624(±0.039)	0.727(±0.050)	0.761(±0.065)	0.1677	0.3756	0.1607	0.8945
2-Isopropylmalate	0.581(±0.039)	0.536(±0.031)	0.645(±0.067)	0.3036	0.8062	0.6288	0.2775
3-Hydroxybutyrate	0.875(±0.061)	0.820(±0.074)	1.021(±0.087)	0.1519	0.8684	0.3504	0.1507
4-Hydroxyphenylacetate	0.555(±0.049)	0.522(±0.041)	0.632(±0.046)	0.2163	0.8698	0.4477	0.2094
Tryptophan	0.824(±0.048)	1.023(±0.059)	0.793(±0.057)	0.0087	0.0359	0.9136	0.0111
Adenine dinucleotide phosphate	0.763(±0.059)	0.653(±0.060)	0.713(±0.057)	0.4337	0.4001	0.8150	0.7529
Allantoin	0.385(±0.029)	0.432(±0.040)	0.472(±0.060)	0.4006	0.7581	0.3667	0.8130
Ascorbic acid	1.031(±0.084)	1.065(±0.097)	0.981(±0.057)	0.7527	0.9523	0.8933	0.7363
Adenosine triphosphate	0.981(±0.066)	1.136(±0.087)	1.221(±0.080)	0.0877	0.3541	0.0743	0.7222
Cholesteryl sulfate	1.031(±0.045)	0.928(±0.070)	0.992(±0.060)	0.4767	0.4487	0.8833	0.7251
Cholic acid	2.151(±0.066)	2.412(±0.185)	2.223(±0.126)	0.3705	0.3579	0.9187	0.5715
Citraconic acid	0.495(±0.047)	0.382(±0.029)	0.417(±0.042)	0.1394	0.1313	0.3501	0.8135
Deoxycholate	0.714(±0.061)	0.889(±0.055)	0.778(±0.067)	0.1475	0.1286	0.7399	0.4205
Fructose 1,6-bisphosphate	1.515(±0.118)	1.664(±0.093)	1.396(±0.093)	0.1856	0.5699	0.6808	0.1588
Fructose 6-phosphate	0.815(±0.066)	0.799(±0.049)	0.844(±0.064)	0.8687	0.9819	0.9378	0.8617
Guanidoacetate	0.797(±0.057)	0.891(±0.078)	0.821(±0.063)	0.5921	0.5845	0.9630	0.7348
Homocysteate	1.061(±0.049)	1.181(±0.078)	1.261(±0.076)	0.1192	0.4526	0.1002	0.6937
Hydroxyisocaproate	0.758(±0.057)	0.711(±0.069)	0.741(±0.088)	0.9043	0.8971	0.9849	0.9554
Kynurenic acid	0.841(±0.063)	0.591(±0.055)	0.801(±0.061)	0.0109	0.0138	0.8833	0.0419
Kynurenine	1.010(±0.046)	0.795(±0.045)	0.981(±0.063)	0.0112	0.0153	0.9172	0.0380
Malate	0.652(±0.048)	0.612(±0.054)	0.721(±0.062)	0.3721	0.8706	0.6449	0.3513
Fumarate	0.861(±0.048)	0.811(±0.066)	0.961(±0.057)	0.1715	0.8168	0.4246	0.1602
Malonyl-CoA	0.879(±0.048)	0.815(±0.068)	0.951(±0.060)	0.2697	0.7321	0.6562	0.2399
Indole 3-carboxylate	0.621(±0.035)	0.778(±0.056)	0.608(±0.036)	0.0107	0.0320	0.9738	0.0163
N-Acetyl-L-Alanine	0.723(±0.039)	0.672(±0.056)	0.697(±0.045)	0.7514	0.7302	0.9161	0.9248
p-Aminobenzoate	1.361(±0.059)	1.296(±0.069)	1.321(±0.067)	0.7824	0.7682	0.8985	0.9604
Phenyllactic	0.792(±0.039)	0.706(±0.054)	0.845(±0.048)	0.1225	0.4229	0.7032	0.1034
Phenylpyruvate	1.287(±0.066)	1.374(±0.054)	1.271(±0.055)	0.4236	0.5595	0.9790	0.4335
Pyroglutamic acid	1.061(±0.049)	1.126(±0.060)	0.987(±0.046)	0.1656	0.6543	0.5564	0.1417
Pyrophosphate	0.538(±0.039)	0.512(±0.037)	0.579(±0.046)	0.5110	0.8996	0.7554	0.4879
Pyruvate	0.716(±0.048)	0.731(±0.054)	0.821(±0.055)	0.3052	0.9787	0.3291	0.4532
Taurine	0.946(±0.057)	0.912(±0.075)	0.933(±0.074)	0.9430	0.9382	0.9901	0.9752
Taurodeoxycholate	0.769(±0.066)	0.924(±0.079)	0.712(±0.055)	0.0734	0.2391	0.8100	0.0667
Uracil	1.001(±0.066)	0.915(±0.078)	1.112(±0.083)	0.1933	0.7150	0.5516	0.1694
Uridine	0.788(±0.057)	0.814(±0.056)	0.721(±0.046)	0.4367	0.9377	0.6353	0.4325
Xanthurenate	1.331(±0.093)	1.251(±0.087)	1.396(±0.091)	0.5307	0.8130	0.8639	0.4986
Acetyl-CoA	1.150(±0.057)	1.190(±0.062)	1.290(±0.065)	0.2491	0.8946	0.2395	0.4912
Adenosine monophosphate	1.437(±0.114)	1.532(±0.127)	1.721(±0.091)	0.1739	0.8210	0.1591	0.4503
Anthranilate	0.745(±0.050)	0.676(±0.059)	0.721(±0.053)	0.6680	0.6487	0.9452	0.8265
Arginosuccinate	0.972(±0.067)	0.998(±0.076)	1.131(±0.090)	0.3035	0.9716	0.3217	0.4632
Biotin	0.990(±0.079)	1.120(±0.090)	1.110(±0.064)	0.4208	0.4739	0.5061	0.9954
Erythrose 4-phosphate	0.935(±0.077)	0.942(±0.074)	1.071(±0.063)	0.3085	0.9974	0.3607	0.4121
Inosine	0.893(±0.040)	1.020(±0.047)	0.912(±0.047)	0.1120	0.1259	0.9502	0.2108
N-carbomyl-aspartate	1.218(±0.095)	1.287(±0.085)	1.120(±0.060)	0.3436	0.8244	0.6603	0.3168
Niacinamide	0.876(±0.046)	0.889(±0.070)	0.791(±0.043)	0.3623	0.9842	0.4871	0.3988
Prephenate	1.341(±0.099)	1.265(±0.089)	1.312(±0.083)	0.8411	0.8287	0.9711	0.9285
Riboflavin 5'-monophosphate	1.242(±0.106)	1.181(±0.082)	1.100(±0.073)	0.5099	0.8801	0.4804	0.7931
Tropisetron	0.935(±0.084)	1.030(±0.085)	1.010(±0.070)	0.6775	0.6852	0.7769	0.9828
Ureidopropionic acid	0.624(±0.052)	0.721(±0.059)	0.699(±0.053)	0.4291	0.4359	0.5867	0.9564
Glucose 6-phosphate	1.150(±0.068)	1.380(±0.115)	1.120(±0.080)	0.0879	0.1740	0.9679	0.1017
Succinate	0.746(±0.059)	0.721(±0.072)	0.926(±0.074)	0.0728	0.9656	0.1518	0.0961
Lactate	0.376(±0.022)	0.421(±0.040)	0.399(±0.035)	0.6418	0.6142	0.8721	0.8862
Citrate	1.190(±0.075)	1.010(±0.692)	0.912(±0.060)	0.8664	0.9411	0.8567	0.9816
Lactulose	0.578(±0.040)	0.514(±0.040)	0.555(±0.040)	0.5414	0.5168	0.9122	0.7551
Sucralose	0.711(±0.055)	0.728(±0.043)	0.810(±0.068)	0.4216	0.9772	0.4372	0.5771
Hippurate	0.699(±0.044)	0.731(±0.062)	0.761(±0.053)	0.7034	0.9076	0.6789	0.9159
Faecal Short Chain Fatty Acids (μM)							
Butyrate	215.000(±23.935)	217.300(±26.098)	456.500(±28.903)	<0.0001	0.9979	<0.0001	<0.0001
Formate	1.340(±0.058)	1.160(±0.064)	1.720(±0.041)	<0.0001	0.0581	<0.0001	<0.0001
Malonate	0.365(±0.039)	0.363(±0.049)	0.500(±0.068)	0.1253	0.9995	0.1847	0.1857
Valerate	30.910(±4.311)	28.510(±4.322)	40.740(±5.703)	0.1220	0.9255	0.2596	0.1341
Propionate	187.500(±16.870)	202.800(±8.179)	245.200(±23.547)	0.0633	0.8265	0.0613	0.2267
Acetate	596.200(±62.093)	548.400(±73.136)	1382.000(±78.082)	<0.0001	0.8891	<0.0001	<0.0001

Supplementary Table 5. Cytokine and chemokine concentration in PBMC culture media following stimulation with NAFLD-HCC, NAFLD-cirrhosis and non-NAFLD control bacterial extract (BE)

Sample size is n=10 non-NAFLD control, n=10 NAFLD-cirrhosis, n=10 NAFLD-HCC as biologically independent samples. Data is shown as mean ± standard error of mean (SEM). *P* values calculated by one-way ANOVA for 3 group comparison and Tukey's post hoc test for 2 group comparisons. $P < 0.05$ considered statistically significant.

Cytokine and chemokine concentration (pg/mL)	non-NAFLD control Mean (±SEM)	NAFLD-cirrhosis Mean (±SEM)	NAFLD-HCC Mean (±SEM)	*P*-value (one-way ANOVA)	*P*-value non-NAFLD control vs NAFLD-cirrhosis	*P*-value non-NAFLD control vs NAFLD-HCC	*P*-value NAFLD-cirrhosis vs NAFLD-HCC
IL-2	0.812(±0.108)	0.832(±0.094)	0.436(±0.116)	0.0225	0.9903	0.0483	0.0361
IL-12 (p70)	1.138(±0.094)	1.388(±0.156)	0.622(±0.134)	0.0010	0.3771	0.0245	0.0008
IL-4	0.852(±0.023)	0.588(±0.019)	0.568(±0.020)	<0.0001	<0.0001	<0.0001	0.7748
IL-6	0.850(±0.217)	0.884(±0.215)	2.060(±0.233)	0.0007	0.9936	0.0018	0.0024
IL-10	0.730(±0.137)	0.760(±0.130)	1.440(±0.177)	0.0030	0.9889	0.0063	0.0090
MIG	0.298(±0.052)	0.376(±0.059)	0.486(±0.076)	0.1250	0.6600	0.1065	0.4435
MIP-1β	0.376(±0.046)	0.266(±0.034)	0.356(±0.033)	0.1154	0.1240	0.9278	0.2380
IFN-α2	0.908(±0.059)	0.686(±0.064)	0.738(±0.070)	0.0549	0.0552	0.1689	0.8372
IFN-γ	2.126(±0.518)	2.220(±0.332)	1.942(±0.273)	0.8766	0.9840	0.9403	0.8692
SDF-1α	0.488(±0.061)	0.424(±0.042)	0.316(±0.054)	0.0866	0.6741	0.0742	0.3358
IL-1ra	1.124(±0.085)	1.134(±0.062)	1.298(±0.047)	0.1361	0.9938	0.1740	0.2090
MCP-3	0.112(±0.012)	0.084(±0.010)	0.098(±0.012)	0.2401	0.2109	0.6650	0.6650
IL-16	0.930(±0.064)	0.742(±0.081)	0.804(±0.071)	0.1922	0.1765	0.4452	0.8180
IL-12 (p40)	1.084(±0.068)	0.992(±0.079)	0.828(±0.082)	0.0748	0.6768	0.0644	0.3013
LIF	0.760(±0.095)	0.532(±0.064)	0.520(±0.091)	0.0969	0.1572	0.1307	0.9945
TNF-β	0.698(±0.101)	0.546(±0.080)	0.498(±0.067)	0.2286	0.4153	0.2268	0.9135
IL-5	0.986(±0.063)	0.932(±0.060)	0.780(±0.053)	0.0529	0.7952	0.0509	0.1811
GM-CSF	0.768(±0.129)	0.502(±0.083)	0.450(±0.071)	0.0632	0.1496	0.0718	0.9247
MIF	0.584(±0.051)	0.520(±0.066)	0.392(±0.087)	0.1572	0.7929	0.1428	0.4053
TNF-α	0.920(±0.083)	0.834(±0.115)	0.778(±0.082)	0.5715	0.7981	0.5459	0.9083
RANTES	0.510(±0.050)	0.380(±0.035)	0.380(±0.040)	0.0565	0.0908	0.0908	>0.9999
IL-1β	1.274(±0.071)	1.196(±0.032)	1.160(±0.070)	0.4093	0.6391	0.3917	0.9078
IL-18	1.064(±0.081)	1.070(±0.054)	0.910(±0.066)	0.1876	0.9979	0.2618	0.2368
Eotaxin	0.882(±0.040)	0.818(±0.047)	0.834(±0.049)	0.5893	0.5845	0.7372	0.9663
Basic FGF	0.890(±0.060)	0.864(±0.040)	0.778(±0.060)	0.3248	0.9385	0.3240	0.5082
VEGF	1.164(±0.027)	1.232(±0.057)	1.240(±0.044)	0.4281	0.5359	0.4610	0.9912
β-NGF	1.042(±0.073)	0.838(±0.067)	0.864(±0.076)	0.1131	0.1320	0.2078	0.9649
PDGF-BB	0.958(±0.031)	1.020(±0.040)	0.900(±0.052)	0.1521	0.5596	0.6008	0.1291
IP-10	0.404(±0.066)	0.466(±0.066)	0.410(±0.068)	0.7716	0.7906	0.9978	0.8253
IL-13	0.701(±0.111)	0.652(±0.034)	0.494(±0.063)	0.1540	0.8932	0.1533	0.3241
MCP-1	0.638(±0.017)	0.574(±0.055)	0.746(±0.063)	0.0599	0.6319	0.2824	0.0504
IL-8	0.902(±0.067)	0.916(±0.043)	0.836(±0.026)	0.4677	0.9771	0.6043	0.4804
MIP-1α	0.472(±0.033)	0.468(±0.032)	0.532(±0.032)	0.3030	0.9957	0.3955	0.3499
GCSF	0.856(±0.020)	0.834(±0.032)	0.848(±0.035)	0.8697	0.8607	0.9803	0.9408
GRO-α	0.662(±0.060)	0.624(±0.061)	0.506(±0.053)	0.1609	0.8895	0.1590	0.3378
HGF	0.890(±0.024)	0.910(±0.038)	0.894(±0.036)	0.9028	0.9042	0.9960	0.9375
IL-1a	0.718(±0.058)	0.624(±0.054)	0.516(±0.078)	0.1008	0.5587	0.0833	0.4664
IL-3	1.176(±0.098)	1.070(±0.066)	0.950(±0.076)	0.1636	0.6309	0.1398	0.5559
SCF	0.830(±0.015)	0.804(±0.044)	0.818(±0.022)	0.8272	0.8119	0.9563	0.9410
TRAIL	0.830(±0.027)	0.790(±0.079)	0.712(±0.049)	0.3305	0.8689	0.3098	0.5911
MCSF	0.602(±0.034)	0.606(±0.031)	0.494(±0.059)	0.1306	0.9976	0.1948	0.1736
CTACK	0.900(±0.047)	0.914(±0.028)	0.868(±0.020)	0.6166	0.9534	0.7808	0.6030
IL-15	0.950(±0.016)	0.958(±0.015)	0.924(±0.022)	0.3918	0.9474	0.5717	0.3901
IL-17	0.768(±0.045)	0.598(±0.064)	0.610(±0.067)	0.0975	0.1268	0.1648	0.9889
IL-9	0.874(±0.044)	0.878(±0.026)	0.710(±0.079)	0.0604	0.9985	0.1012	0.0913
SCGF-β	0.450(±0.071)	0.430(±0.054)	0.318(±0.027)	0.1928	0.9626	0.2105	0.3194
