# Supplementary material for: Gut microbiota impact on the peripheral immune response in non-alcoholic fatty liver disease related hepatocellular carcinoma
Source: Nat Commun. 2021 Jan 8;12:187. doi: 10.1038/s41467-020-20422-7 (PMC7794332; doi:10.1038/s41467-020-20422-7)
Supplement: Supplementary file 3 — Reporting Summary [file 41467_2020_20422_MOESM3_ESM.pdf]

nature research

Corresponding author(s): A/Prof Amany Zekry

Last updated by author(s): Nov 4, 2020

Reporting Summary

Nature Research wishes to improve the reproducibility of the work that we publish. This form provides structure for consistency and transparency in reporting. For further information on Nature Research policies, see our Editorial Policies and the Editorial Policy Checklist.

Statistics

For all statistical analyses, confirm that the following items are present in the figure legend, table legend, main text, or Methods section.

n/a	Confirmed	
☐	☒	The exact sample size (n) for each experimental group/condition, given as a discrete number and unit of measurement
☐	☒	A statement on whether measurements were taken from distinct samples or whether the same sample was measured repeatedly
☐	☒	The statistical test(s) used AND whether they are one- or two-sided<br>*Only common tests should be described solely by name; describe more complex techniques in the Methods section.*
☐	☒	A description of all covariates tested
☐	☒	A description of any assumptions or corrections, such as tests of normality and adjustment for multiple comparisons
☐	☒	A full description of the statistical parameters including central tendency (e.g. means) or other basic estimates (e.g. regression coefficient) AND variation (e.g. standard deviation) or associated estimates of uncertainty (e.g. confidence intervals)
☐	☒	For null hypothesis testing, the test statistic (e.g. F, t, r) with confidence intervals, effect sizes, degrees of freedom and P value noted<br>*Give P values as exact values whenever suitable.*
☒	☐	For Bayesian analysis, information on the choice of priors and Markov chain Monte Carlo settings
☒	☐	For hierarchical and complex designs, identification of the appropriate level for tests and full reporting of outcomes
☐	☒	Estimates of effect sizes (e.g. Cohen's d, Pearson's r), indicating how they were calculated

Our web collection on statistics for biologists contains articles on many of the points above.

Software and code

Policy information about availability of computer code

Data collection	No custom computer code was used for data collection.<br>Patient meta-data was recorded in Microsoft Excel (v16.41).<br>Metabolite data was collected with NMR Suite Professional (v8.2) (Chenomx Inc., Edmonton, AB, Canada).<br>Cytokine data was collected with Bio-Plex Manager Software (v6.0).<br>Flow cytometry data was collected with BD FACSDiva(TM) software (v8.0.3) (BD Biosciences).
Data analysis	No custom computer code was used for data analysis.<br>Prior to analysis of microbiome data, removal of low quality reads was performed with fastp (v0.19.5) (https://github.com/OpenGene/fastp). Dereplicate of metagenomic sequences was performed with BBmap (v38.79-0) (https://sourceforge.net/projects/bbmap/). Removal of host decontamination was performed with minimap2 (v2.16) (https://github.com/lh3/minimap2). Microbiome compositional profiling was performed using KrakenUniq (v0.5.8) (https://github.com/fbreitwieser/krakenuniq). Differential abundance analysis was performed with LEfSe (v1.0) (https://github.com/SegataLab/lefse) and capscale function in Vegan2 (v2.5-6) (https://cran.r-project.org/web/packages/vegan/).<br>Microbiome function was annotated with HUMAnN2 (v2.0) (https://github.com/biobakery/humann)<br>Microbiome analysis was completed in R (v3.6.1).<br>Flow cytometry data analysis was performed with FlowJo software (v10.5.3) (TreeStar).<br>Metabolite, cytokine and PBMC analysis and data visualization was performed in Prism (v8.2.1) (GraphPad Software, Inc.).<br>Correlation analysis was performed in R (v3.6.1).

For manuscripts utilizing custom algorithms or software that are central to the research but not yet described in published literature, software must be made available to editors and reviewers. We strongly encourage code deposition in a community repository (e.g. GitHub). See the Nature Research guidelines for submitting code & software for further information.

Data

Policy information about availability of data

All manuscripts must include a data availability statement. This statement should provide the following information, where applicable:

- Accession codes, unique identifiers, or web links for publicly available datasets
- A list of figures that have associated raw data
- A description of any restrictions on data availability

All metagenomics data has been uploaded to the National Center for Biotechnology Information (NCBI) (https://www.ncbi.nlm.nih.gov/), accession code: PRJNA647523.
Following functional annotation, microbiota function was transformed into the Kyoto Encyclopedia of Genes and Genomes (KEGG) Orthologies (PubMed:16381885) [DOI:10.1093/nar/gkj102).
The authors declare that data supporting the findings of this study are available within the paper (and its Supplementary Information files).

Field-specific reporting

Please select the one below that is the best fit for your research. If you are not sure, read the appropriate sections before making your selection.

[x] Life sciences [ ] Behavioural & social sciences [ ] Ecological, evolutionary & environmental sciences

For a reference copy of the document with all sections, see nature.com/documents/nr-reporting-summary-flat.pdf

Life sciences study design

All studies must disclose on these points even when the disclosure is negative.

Sample size	A total of 90 subjects were recruited in the study; 32 with NAFLD-HCC, 28 with NAFLD-cirrhosis and 30 non-NAFLD control. No formal sample-size calculation was performed. Rather, our sample size was estimated based on previous publications demonstrating statistical difference in similar outcome measures (microbial diversity and inflammatory/immune responses) in comparable patient cohorts (Ponziani FR, et al. Hepatocellular Carcinoma Is Associated With Gut Microbiota Profile and Inflammation in Nonalcoholic Fatty Liver Disease. Hepatology. 2019 Jan;69(1):107-120).
Data exclusions	No data was excluded from the analysis.
Replication	All samples were measured once as biologically independent samples.
Randomization	Randomization was not required as this was not a clinical study or clinical trial, but rather subjects were prospectively recruited for the key purpose of sample collection as required for the described microbiome and immune studies.
Blinding	Blinding was not required as this was not a clinical study or clinical trial. Rather, patients were recruited for the key purpose of sample collection and hence knowledge of disease phenotype for sample identification was essential.

Reporting for specific materials, systems and methods

We require information from authors about some types of materials, experimental systems and methods used in many studies. Here, indicate whether each material, system or method listed is relevant to your study. If you are not sure if a list item applies to your research, read the appropriate section before selecting a response.

Materials & experimental systems

n/a	Involved in the study	
[ ]	[x]	Antibodies
[x]	[ ]	Eukaryotic cell lines
[x]	[ ]	Palaeontology and archaeology
[x]	[ ]	Animals and other organisms
[ ]	[x]	Human research participants
[x]	[ ]	Clinical data
[x]	[ ]	Dual use research of concern

Methods

n/a	Involved in the study	
[x]	[ ]	ChIP-seq
[ ]	[x]	Flow cytometry
[x]	[ ]	MRI-based neuroimaging

Antibodies

Antibodies used	Anti-human CD3 (BD Biosciences, #555336, Lot. 7312853), Anti-human CD28 (BD Biosciences, #555725, Lot. 8152601), Anti-human IL-4 (BD Biosciences #554605, Lot. 8110721), Anti- Annexin V-BV605 (BD Biosciences, #563974, Lot.8282753), Anti- CD3-PE-Cy7 (BD Biosciences, #563423, Lot. 9079612), Anti- CD4-PerCP¬Cy5.5 (BD Biosciences, #560650, Lot. 4324277), Anti- CD25-BV421 (BD Biosciences, #562442, Lot. 8331556), Anti-Foxp3-AF488 (BD Biosciences, #560047, Lot. 8157861), Anti- IL-10-PE (BD Biosciences,

#559337, Lot. 7118693), Anti- CD8-AP7-H7 (BD Biosciences, #560179, 9108663), Anti- CD45RO-BV711 (BD Biosciences, #563722, Lot. 9009651), Anti- CCR7-PE (BD Biosciences, #560765, Lot. 9025622), Anti- CD14-APC (BD Biosciences, #555399, Lot. 7304714), Anti-CD11c-PE (BD Biosciences, #555392, Lot.8298946), Anti- HLA-DR-APC-H7 (BD Biosciences, #561358, Lot. 8318843), Anti- CD19-BB515 (BD Biosciences, #564456, Lot. 8248828), Anti- CD20-BB515 (BD Biosciences, #564568, Lot. 9064592), Anti- T-bet-PE (BD Biosciences, #561265, Lot. 8142784).

Validation

All antibodies used in experiments are commercially available and have been validated by the manufacturer. FACS plots can be found by accessing the website indicated below. Additionally, all antibodies have been validated for use in lymphocytes as indicated below, where possible.
Anti-human CD3 (BD Biosciences, #555336, Lot. 7312853), https://www.bdbiosciences.com/anz/applications/research/t-cell-immunology/th-1-cells/surface-markers/human/purified-nale-mouse-anti-human-cd3-hit3a/p/555336.
Anti-human CD28 (BD Biosciences, #555725, Lot. 8152601), https://www.bdbiosciences.com/anz/applications/research/t-cell-immunology/regulatory-t-cells/surface-markers/human/purified-nale-mouse-anti-human-cd28-cd282/p/555725.
Anti-human IL-4 (BD Biosciences #554605, Lot. 8110721), https://www.bdbiosciences.com/anz/applications/research/t-cell-immunology/th-2-cells/immunoassays/elisa/human/recombinant-human-il-4/p/554605.
Anti- Annexin V-BV605 (BD Biosciences, #563974, Lot. 8282753), https://www.bdbiosciences.com/anz/reagents/research/cell-based-assays/apoptosis/bv605-annexin-v/p/563974, DOI: 10.1016/0022-1759(95)00072-I.
Anti- CD3-PE-Cy7 (BD Biosciences, #563423, Lot. 9079612), https://www.bdbiosciences.com/anz/applications/research/t-cell-immunology/th-1-cells/surface-markers/human/pe-cy7-mouse-anti-human-cd3-ucht1-also-known-as-ucht-1-ucht-1/p/563423, http://www.jimmunol.org/content/133/1/129.
Anti- CD4-PerCP¬Cy5.5 (BD Biosciences, #560650, Lot. 4324277), https://www.bdbiosciences.com/anz/applications/research/stem-cell-research/hematopoietic-stem-cell-markers/human/negative-markers/percp-cy55-mouse-anti-human-cd4-rpa-t4/p/560650.
Anti- CD25-BV421 (BD Biosciences, #562442, Lot. 8331556), https://www.bdbiosciences.com/anz/applications/research/t-cell-immunology/regulatory-t-cells/surface-markers/human/bv421-mouse-anti-human-cd25-m-a251/p/562442.
Anti- Foxp3-AF488 (BD Biosciences, #560047, Lot. 8157861), https://www.bdbiosciences.com/anz/applications/research/t-cell-immunology/regulatory-t-cells/intracellular-markers/cell-signalling-and-transcription-factors/human/alexa-fluor-488-mouse-anti-human-foxp3-259dc7/p/560047, DOI: 10.1002/eji.200526189.
Anti- IL-10-PE (BD Biosciences, #559337, Lot. 7118693), https://www.bdbiosciences.com/anz/applications/research/b-cell-research/intracellular-antigens/human/pe-rat-anti-human-and-viral-il-10-jes3-9d7/p/559337.
Anti- CD8-AP7-H7 (BD Biosciences, #560179, 9108663), https://www.bdbiosciences.com/anz/reagents/research/antibodies-buffers/immunology-reagents/anti-human-antibodies/cell-surface-antigens/apc-h7-mouse-anti-human-cd8-sk1/p/560179, DOI: 10.1007/978-3-642-68857-7_66.
Anti- CD45RO-BV711 (BD Biosciences, #563722, Lot. 9009651), https://www.bdbiosciences.com/anz/applications/research/t-cell-immunology/regulatory-t-cells/surface-markers/human/bv711-mouse-anti-human-cd45ro-uchl1/p/563722.
Anti- CCR7-PE (BD Biosciences, #560765, Lot. 9025622), https://www.bdbiosciences.com/anz/applications/research/t-cell-immunology/th-2-cells/surface-markers/human/pe-mouse-anti-human-cd197-ccr7-150503/p/560765, DOI: 10.1074/jbc.273.12.7118.
Anti- CD14-APC (BD Biosciences, #555399, Lot. 7304714), https://www.bdbiosciences.com/anz/applications/research/stem-cell-research/hematopoietic-stem-cell-markers/human/negative-markers/apc-mouse-anti-human-cd14-m5e2/p/555399, DOI: 10.1126/science.1698311.
Anti- CD11c-PE (BD Biosciences, #555392, Lot. 8298946), https://www.bdbiosciences.com/anz/reagents/research/antibodies-buffers/immunology-reagents/anti-human-antibodies/cell-surface-antigens/pe-mouse-anti-human-cd11c-b-ly6/p/555392
Anti- HLA-DR-APC-H7 (BD Biosciences, #561358, Lot. 8318843), https://www.bdbiosciences.com/anz/applications/research/stem-cell-research/mesenchymal-stem-cell-markers-bone-marrow/human/negative-markers/apc-h7-mouse-anti-human-hla-dr-g46-6/p/561358, https://doi.org/10.1084/jem.193.11.1303
Anti- CD19-BB515 (BD Biosciences, #564456, Lot. 8248828), https://www.bdbiosciences.com/anz/applications/research/stem-cell-research/hematopoietic-stem-cell-markers/human/negative-markers/bb515-mouse-anti-human-cd19-hib19/p/564456.
Anti- CD20-BB515 (BD Biosciences, #564568, Lot. 9064592), https://www.bdbiosciences.com/anz/reagents/research/antibodies-buffers/immunology-reagents/anti-human-antibodies/cell-surface-antigens/bb515-mouse-anti-human-cd20-2h7/p/564568, DOI: 10.1002/cyto.990140212.
Anti- T-bet-PE (BD Biosciences, #561265, Lot. 8142784), https://www.bdbiosciences.com/anz/applications/research/t-cell-immunology/th-1-cells/intracellular-markers/cell-signalling-and-transcription-factors/human/pe-mouse-anti-t-bet-4b10/p/561265, DOI: 10.1016/S0092-8674(00)80702-3.

Human research participants

Policy information about studies involving human research participants

Population characteristics	Population characteristics of patients recruited into the study are described in Tables 1 and 2 of the manuscript.<br>Covariates in Table 1: demographic data (age, gender, BMI), clinical data (transient elastography score, Child Pugh Score, MELD-Na Score, varices), co morbidity data (type II diabetes, metformin use, essential hypertension, dyslipidaemia, diet) and biochemical data (ALT, AST, platelet count, albumin, INR).<br>Covariates in Table 2: tumour number, tumour size, BCLC stage, histology confirming cirrhosis, histology of tumour (grade), hepatic venous pressure gradient, alpha-fetoprotein (tumour marker) level.
Recruitment	Three groups were consecutively recruited to the study. These were: 1) Subjects with NAFLD (non-alcoholic fatty liver disease) cirrhosis related hepatocellular carcinoma (NAFLD-HCC) who were undergoing surgical resection; 2) Subjects with NAFLD related liver cirrhosis (NAFLD-cirrhosis) and 3) non-NAFLD controls.<br><br>NAFLD was diagnosed based on the American Association for the Study of Liver Diseases practice guidelines. Liver cirrhosis was confirmed based on clinical, biochemical, transient elastography, and radiological assessment in the NAFLD-cirrhosis group; and additionally, confirmed on histology in resection specimens in the NAFLD-HCC group. HCC was diagnosed according to international guidelines, integrating history, physical examination, biochemistry, and imaging techniques obtained by multiphasic CT, and/or dynamic contrast-enhanced MRI. Diagnosis of HCC was further confirmed by histopathological examination of surgical resection specimens. Treatment decisions for HCC were determined in multidisciplinary meetings, based on international guidelines. For non-NAFLD controls, a detailed medical history was taken including medications that could lead to steatosis. All participants underwent a physical examination and had routine blood tests (including liver function tests, serological tests for hepatitis B surface antigen, hepatitis C virus antibody, autoimmune screen, and iron studies) in addition to a screening liver ultrasound to exclude hepatic steatosis.<br><br>For the groups with liver cirrhosis, exclusion criteria included: subjects aged < 18 years; alcohol consumption > 30g daily for men and > 20g daily for women, other causes of liver cirrhosis (including viral, alcoholic, autoimmune, cholestatic liver diseases and inherited liver diseases etc); previous clinical or biochemical evidence of hepatic decompensation; any degree of portal hypertension (based on clinical, radiological and endoscopy or pre-operative hepatic venous pressure gradient (HVPG)); other primary liver cancers (e.g. mixed hepatocellular carcinoma and cholangiocarcinoma); probiotic or antibiotic administration (per oral or intravenous) in the 3 months prior to recruitment; known gastrointestinal disease; previous gastrointestinal surgery; regular proton-pump inhibitor or lactulose therapy. For non-NAFLD controls, exclusion criteria included: subjects aged < 18 years; alcohol consumption 30g daily for men and 20g daily for women, known history of liver disease (including viral, alcoholic, autoimmune, cholestatic liver diseases and inherited liver diseases), liver ultrasound demonstrating hepatic steatosis, known gastrointestinal disease, previous gastrointestinal surgery, regular proton-pump inhibitor, lactulose therapy or antibiotic/probiotic administration (per oral or intravenous) in the 3 months prior to recruitment.<br><br>Although selection bias is possible in this study; the population characteristics of our cohort are representative of the Australian population with NAFLD-cirrhosis and NAFLD-HCC. (Mahady, S. E., and Adams, L. A. (2018) Burden of non-alcoholic fatty liver disease in Australia. Journal of Gastroenterology and Hepatology, 33: 1– 11. doi: 10.1111/jgh.14270)
Ethics oversight	The study was approved by Sydney Local Health District Human Research Ethics Committee (HREC), Sydney Local Health District (SLHD), NSW Health with local governance approval (HREC/16/RPAH/701; SSA18/G/058). Informed consent was obtained from all study participants.

Note that full information on the approval of the study protocol must also be provided in the manuscript.

Flow Cytometry

Plots

Confirm that:

- [x] The axis labels state the marker and fluorochrome used (e.g. CD4-FITC).
- [x] The axis scales are clearly visible. Include numbers along axes only for bottom left plot of group (a 'group' is an analysis of identical markers).
- [x] All plots are contour plots with outliers or pseudocolor plots.
- [x] A numerical value for number of cells or percentage (with statistics) is provided.

Methodology

Sample preparation	Peripheral blood mononuclear cells (PBMCs) were isolated from non-NAFLD control participants. Following blood sample collection, 10 mL of whole blood was centrifuged at 700 xg for 10 min. Plasma was removed, and PBS added to volume of 30 mL. Next, 15mL of Ficoll® solution (Merck, #26873-85-8) was gently underlayed with a mixing cannula, and centrifuged at 700 xg for 25 min at room temperature. PBMCs were harvested from the Ficoll® separation layer; cell count and viability were assessed with light microscopy using 20 uL of PBMC sample mixed with 20 uL of 0.4% trypan blue solution (ThermoFisher, #15250061). PBMCs were resuspended in 10% DMSO in RPMI media supplemented with autologous plasma and stored in liquid nitrogen for downstream experiments. PBMCs were seeded in flat-bottom culture plates (106 cells/mL) and incubated with RPMI media supplemented with 10% FBS and 1% penicillin/streptomycin (Life technologies, #15140122) and 1% glutamine (Life Technologies, #35050061). For human Treg differentiation, PBMCs were stimulated with anti-human CD3 (0.3

µg/mL; BD Biosciences, #555336), anti-human CD28 (2 µg/mL; BD Biosciences, #555725) and recombinant human TGF-β1 (2.5ng/mL; R&D Systems #240B002) for 3 days in presence of BE. For human Th1 differentiation, PBMCs were stimulated with anti-human CD3 (2 µg/mL; BD Biosciences #555336), anti-human CD28 (2 µg/mL; BD Biosciences, #555725), anti-human IL-4 (5 µg/mL; BD Biosciences #554605) and recombinant human IL-12 (20ng/mL; BD Biosciences #554613) for 3 days in presence of BE. After 3 days the culture media was changed and Th1 cells were restimulated for 4 hours with ionomycin (1µM; Sigma-Aldrich, #I3909) and PMA (50ng/ml; Sigma-Aldrich, #P1585) in presence of monensin (2 µM; Life Technologies, #00-4505-51). Undifferentiated PBMCs were stimulated for 3 days in the presence of BE. Cells were harvested and centrifuged at 300 xg for 5 mins. The supernatant was removed, followed by resuspension in 1 mL of PBS. Cell count and viability were again assessed with light microscopy using 20 uL of PBMC sample mixed with 20 uL of 0.4% trypan blue solution (ThermoFisher, #15250061).

Instrument

Flow cytometry was performed on BD LSRFortessa (TM) X-20 Cell Analyzer (BD Biosciences, #657675R1)

Software

Collection of flow cytometry data was performed by BD FACSDiva(TM) software (v8.0.3) (BD Biosciences). Flow cytometry data analysis was performed with FlowJo software (v10.5.3) (TreeStar).

Cell population abundance

FACS analysis was performed on each sample to a total cell number of 50,000 events with a threshold of 10,000 to increase quality of samples per event. FACS quality was also ensured using compensation controls to verify that observed populations were accurate and distinct.

Gating strategy

A detailed gating strategy figure for all target cell populations is provided in Supplementary Information.
1) Effector and IL-10+ Regulatory T cells: SSC-A/FSC-A for lymphocytes, SSC-A/Viability 510 (neg) for viable cells, SSC-A/CD3+ for CD3+ T lymphocytes, CD8+/CD4+ for CD8+ and CD4+ lymphocytes, from CD4+ Foxp3+/CD25+ for regulatory T cells (Tregs), SSC-A/CD45RO+ for effector Tregs, SSC-A/IL-10+ for IL-10+ effector regulatory T cells (Tregs)
2) CD8+ and cytotoxic CD8+ T cells: SSC-A/FSC-A for lymphocytes, SSC-A/Viability 510 (neg) for viable cells, SSC-A/CD3+ for CD3+ T lymphocytes, CD8+/CD4- for CD8+ lymphocytes, CCR7-/CD45RO- for cytotoxic CD8+ T cells
3) CD4+ and CD4+ T-helper cells: SSC-A/FSC-A for lymphocytes, SSC-A/Viability 510 (neg) for viable cells, SSC-A/CD3+ for CD3+ T lymphocytes, CD8-/CD4+ for CD4+ lymphocytes, from CD4+ SSC-A/Tbet+ for Th1 T cells
4) B cells, Monocytes and Dendritic cells: SSC-A/SSC-H for singlets, SSC-A/Viability 510 (neg) for viable cells, SSC-A/CD3- for non-T lymphocytes, SSC-A/CD19/20+ for B cells, SSC-A/CD14+ for monocytes and from CD14- SSC-A/HLADR2+ for dendritic cells.

☒ Tick this box to confirm that a figure exemplifying the gating strategy is provided in the Supplementary Information.
